# Single-cell transcriptomics reveals subtype-specific molecular profiles in Nrf2-deficient macrophages from murine atherosclerotic aortas

**DOI:** 10.3389/fimmu.2023.1249379

**Published:** 2023-10-27

**Authors:** Katarzyna Sarad, Monika Stefańska, Izabela Kraszewska, Krzysztof Szade, Judith C. Sluimer, Przemysław Błyszczuk, Józef Dulak, Agnieszka Jaźwa-Kusior

**Affiliations:** ^1^ Department of Medical Biotechnology, Faculty of Biochemistry, Biophysics and Biotechnology, Jagiellonian University, Kraków, Poland; ^2^ Jagiellonian University, Doctoral School of Exact and Natural Sciences, Kraków, Poland; ^3^ Department of Clinical Immunology, Jagiellonian University Medical College, Kraków, Poland; ^4^ Laboratory of Stem Cell Biology, Faculty of Biochemistry, Biophysics and Biotechnology, Jagiellonian University, Kraków, Poland; ^5^ Cardiovascular Research Institute Maastricht (CARIM), Department of Pathology, Maastricht University Medical Center (UMC), Maastricht, Netherlands; ^6^ BHF Centre for Cardiovascular Science, University of Edinburgh, Edinburgh, United Kingdom; ^7^ Department of Rheumatology, University Hospital Zurich, University of Zurich, Zurich, Switzerland

**Keywords:** atherosclerosis, scRNA-seq, Nrf2, macrophages, heterogeneity, monocytes

## Abstract

Nuclear factor erythroid 2-related factor 2 (Nrf2) is a transcriptional regulator of antioxidant and anti-inflammatory response in all cell types. It also activates the transcription of genes important for macrophage function. Nrf2 activity declines with age and has been closely linked to atherosclerosis, but its specific role in this vascular pathology is not clear. Atherosclerotic plaques contain several macrophage subsets with distinct, yet not completely understood, functions in the lesion development. The aim of this study was to analyze the transcriptome of diverse Nrf2-deficient macrophage subpopulations from murine atherosclerotic aortas. Mice with transcriptionally inactive Nrf2 in Cdh5-expressing cells (*Nrf2* ^Cdh5tKO^) were used in the experiments. These mice lack transcriptional Nrf2 activity in endothelial cells, but also in a proportion of leukocytes. We confirmed that the bone marrow-derived and tissue-resident macrophages isolated from *Nrf2* ^Cdh5tKO^ mice exhibit a significant decline in Nrf2 activity. Atherosclerosis was induced in *Nrf2* ^Cdh5tKO^ and appropriate control mice *via* adeno-associated viral vector (AAV)-mediated overexpression of murine proprotein convertase subtilisin/kexin type 9 (Pcsk9) in the liver and high-fat diet feeding. After 21 weeks, live aortic cells were sorted on FACS and single-cell RNA sequencing (scRNA-seq) was performed. Unsupervised clustering singled out 13 distinct aortic cell types. Among macrophages, 9 subclusters were identified. Differential gene expression analysis revealed cell subtype-specific expression patterns. A subset of inflammatory macrophages from atherosclerotic *Nrf2* ^Cdh5tKO^ mice demonstrated downregulation of DNA replication genes (e.g. *Mcm7*, *Lig1*, *Pola1*) concomitant with upregulation of DNA damage sensor *Atr* gene. Atherosclerotic *Nrf2* ^Cdh5tKO^ Lyve1+ resident macrophages showed strong upregulation of IFN-stimulated genes, as well as changes in the expression of death pathways-associated genes (*Slc40a1*, *Bcl2a1*). Furthermore, we observed subtype-specific expression of core ferroptosis genes (e.g. *Cp, Hells*, *Slc40a1*) in inflammatory *versus* tissue resident macrophages. This observation suggested a link between ferroptosis and inflammatory microenvironment appearing at a very early stage of atherogenesis. Our findings indicate that *Nrf2* deficiency in aortic macrophages leads to subtype-specific transcriptomic changes associated with inflammation, iron homeostasis, cell injury or death pathways. This may help understanding the role of aging-associated decline of Nrf2 activity and the function of specific macrophage subtypes in atherosclerotic lesion development.

## Introduction

Atherosclerosis is an inflammatory disease of the wall of large- and medium-sized arteries. It can begin in childhood with the development of fatty streaks due to an accumulation of lipids in the intimal layer of the artery (1). With time, the fatty streak evolves into established plaque with the thick fibrous cap produced by vascular smooth muscle cells. Then, increasing lipid content and numbers of inflammatory macrophages, enlarged necrotic core and thinner fibrous cap lead to the formation of unstable plaque and sometimes its rupture. That end stage disease is typical for humans, but practically does not occur in mouse models of atherosclerosis (2).

Inflammation and oxidative stress are crucial elements in progression of atherosclerosis. In fact, these two processes are interrelated and form a vicious circle during atherogenesis. Reactive oxygen species induce the expression of inflammatory cytokines, chemokines and soluble mediators of inflammation *via* activation of various transcription factors (3). In turn, cytokines and chemokines produced by inflammatory cells recruit additional inflammatory cells to the sites of inflammation, what increases oxidative stress and exacerbates this adverse cycle.

Macrophages are the most numerous immune cells in the pathogenesis of atherosclerosis, present through all stage of the disease from lesion initiation to plaque rupture (4). Lipoproteins sequestered by macrophages in the arterial wall undergo various modifications including oxidation and aggregation. These pro-inflammatory particles lead to activation of the overlying endothelial cells and recruitment of monocytes differentiating to macrophages inside the vessel wall (5). However, lesional macrophages accumulate not only through recruitment and differentiation of circulating monocytes, but also *via* local proliferation, (trans)-differentiation of vascular smooth muscle cells or local progenitors (4). During atherosclerosis, macrophages are exposed to many environmental signals, which modulate their functional phenotypes. For e.g. cholesterol crystals, found not only in advanced plaques, but also at early stages of atherosclerotic lesions, were shown to activate the caspase-1-activating-NLRP3 inflammasome and thus were shown to act as a proinflammatory stimulus in LPS-primed human peripheral blood mononuclear cells in culture and *in vivo* in mice (6).

Recent research involving the single-cell transcriptomics identified several distinct macrophage clusters within human and mouse atherosclerotic plaques (7–9). These populations may play either beneficial or harmful functions in atherosclerosis, which may depend on the stage of the disease (10, 11). Undeniably, the beneficial role of plaque macrophages, especially at the early stage of the lesion development, results from their ability to scavenge cytotoxic lipoproteins and remove dead cells. But, similarly to other cells in the plaque, macrophages also undergo different forms of cell death. It is currently proposed that targeting the diverse types of macrophage death may affect different stages of atherosclerosis development (12).

Nuclear factor erythroid 2-related factor 2 (Nrf2), a transcription factor encoded in humans by the *NFE2L2* gene, is regarded as a master transcriptional regulator of cellular redox homeostasis. Nrf2 induces the expression of a battery of genes involved in defence against oxidative stress (13). This transcription factor is ubiquitously expressed in vascular cells, where it regulates expression of various atheroprotective enzymes, such as heme oxygenase-1 (14) or peroxiredoxin-1 (15). Moreover, Nrf2 is involved in regulation of iron metabolism. It may affect the labile iron pool *via* regulation of the expression of ferroportin, an iron transporter (16, 17) and ferritin, an iron storage protein (18). Nrf2 also plays an important role in biosynthesis and degradation of iron-containing heme *via* regulation of the expression of genes such as ATP binding cassette subfamily B member 6 or ferrochelatase (19), as well as the above-mentioned heme degrading enzyme - heme oxygenase-1 (14). Noteworthy, Nrf2 activity declines with age what may lead to disturbed redox homeostasis, cell senescence and even cell death (18, 20, 21).

The data on the role of Nrf2 in atherosclerosis are contradictory. Nrf2 was shown to confer protection against foam cells formation by regulating the expression of antioxidant proteins and scavenger receptors in bone marrow transplantation model (22). However, a decreased susceptibility to apolipoprotein E (ApoE)-mediated atherosclerotic plaque formation was shown in Nrf2^−/−^ApoE^−/−^ mice (23, 24). On the other hand, increased atherosclerosis in low-density lipoprotein receptor–deficient (Ldlr^–/–^) mice was observed following Nrf2^−/−^ bone marrow transplantation (25). Another study demonstrated that the ablation of Nrf2 in the bone marrow-derived cells suppresses atherosclerotic lesion area (26). More recently, global Nrf2 deficiency was shown to promote the signs of plaque instability in Ldlr^−/−^ApoB^100/100^ mice *via* increased inflammation and oxidative stress (27).

The contradictory data on the role of Nrf2 in atherosclerosis may result from different approaches in atherosclerosis induction, the genetic background of mice and/or a combination of systemic and local effects in conventional Nrf2 knockout mice. Thus, it is crucial to investigate the association between Nrf2 signaling and fate of specific vascular cell types in atherosclerosis. In this study we aimed to analyze at the single-cell level the transcriptome of diverse macrophage subpopulations from murine atherosclerotic aortas in the early stages of the disease development. Our data indicate that decreased Nrf2 transcriptional activity affects expression of genes involved in inflammatory pathways, cell proliferation and programmed cell death in a subtype-specific manner.

## Materials and methods

### Animals

All mouse experiments were carried out in accordance with Directive 2010/63/UE of the European Parliament on the protection of animals used for scientific purposes and approved by the 2nd Institutional Animal Care and Use Committee (IACUC) in Kraków, Poland (approval number 142/2020). The animals were housed in specific pathogen-free (SPF) conditions with water and food available ad libitum. To generate mice with Nrf2 transcriptional knockout in Cdh5-expressing cells (Nrf2^Cdh5tKO^) and control mice (Nrf2^flox/flox^), C57BL/6-Nfe2l2^tm1.1Sred^/SbisJ mice (The Jackson Laboratory, Strain #:025433) were crossed with B6;129-Tg(Cdh5-cre)1Spe/J mice (The Jackson Laboratory, Strain #: 017968). Genotyping of animals was performed by PCR on the DNA isolated from the tail tips.

### AAV8-Pcsk9 vector production

Production of the adeno-associated viral vector serotype 8 coding for proprotein convertase subtilisin/kexin type 9 (AAV8-Pcsk9) was performed using CellRoll Roller Bottle system (Integra) in three-plasmid Helper-free system as previously described (25). Briefly, AAV293 cells seeded on collagen-precoated Roller Bottles (Corning) were cultured until 60% confluence and transfected with 130 µg of pHelper (Stratagene), 100 µg of pAAV2/8 (kindly provided by Prof. James Wilson, University of Pennsylvania), and 90 µg of pAAV/D377Y-mPCSK9. The plasmid encoding murine Pcsk9 under the control of liver-dependent promoter (pAAV/D377Y-mPCSK9) was a gift from Jacob Bentzon (Addgene plasmid # 58376; http://n2t.net/addgene:58376; RRID: Addgene_58376). According to Addgene specification, the plasmid possessed an alternate 5’ITR sequence which, in our hands, prevented AAV production. Thus, to replace the alternate 5’ITR with the functional 5’ITR, the pAAV-MCS (Stratagene) and pAAV/D377Y-mPCSK9 were digested with Not1 (New England Biolabs) according to the vendor’s protocol. The fragment encoding functional 5’ITR from pAAV-MCS (backbone) and the fragment with Pcsk9 coding gene from pAAV/D377Y-mPCSK9 (GOI) were excised from 1% agarose gel and purified using Zymoclean Gel DNA Recovery Kit (ZymoResearch). Then the backbone was dephosphorylated and ligated with GOI. The proper modified plasmid sequence was confirmed by restriction analysis and functional tests. AAV293 cell transfection was performed using PEI MAX (2.58 mg/ml, 1 µl per 1 µg of DNA). Then, 72 hours after transfection, the cells were detached and washed with PBS. The cells were suspended in a small volume (2-3 ml) of PBS containing calcium and magnesium and subjected to 3 freeze-thaw cycles by placing them in liquid nitrogen and thawing in a 37°C water bath with vigorous mixing after each cycle. The lysate was incubated with HS nuclease (50 U/ml; MoBiTec) for 1 hour at 37°C. Then lysate was centrifuged (4000 *× g*, 30 min, 4°C) twice, the supernatant was collected and stored at -20°C. Next, AAV8-Pcsk9 vector was purified by Iodixanol (OptiPrep™, Sigma-Aldrich) gradient ultracentrifugation as previously described (28).

The AAV8-Pcsk9 titer was determined using quantitative PCR (qPCR). Briefly, the DNA was isolated from 5 µl of stock using the phenol-chloroform extraction method. Quantification of genome copies in the sample was performed using TaqMan-quantitative real-time PCR. The reaction was performed using primers recognizing the sequence of promoter (HCR_F: 5’-TGGAGTGCAGTGACACAATC-3’, HCR_R: 5’-AGGCCTGTAATCCCAGTTAC-3’), the custom Taqman probe (HCR_probe: 5’-6-FAM-GTAGCTGGGATTACAAGCATGTGC-BHQ-1-3’) and TaqMan Gene Expression Master Mix (Applied Biosystems). The serial dilutions of linearized modified pAAV/D377Y-mPCSK9 were used to generate a standard curve. The reaction conditions were applied according to the manufacturer’s protocol. The AAV titer was calculated as the number of vector genomes per µl of purified stock vector preparation.

### Atherosclerosis model

The experiment started when the mice were 3-month-old. To induce hyperlipidemia and atherosclerosis, male Nrf2^Cdh5tKO^ and Nrf2^flox/flox^ mice received a single tail vein injection of AAV8-Pcsk9 at a dose of 1×10^11^ vector genomes (vg) per mouse and were subsequently placed on a high-fat diet (35% fat; 60% kcal from fat; ZooLab, DP-1E-60S) for 21 weeks. Normocholesterolemic 3-month-old male Nrf2^Cdh5tKO^ and Nrf2^flox/flox^ control mice received a single tail vein injection of saline and were fed a standard diet. At the end of the experiment, the mice were euthanized by inhalation of an overdose of isoflurane. Next, the blood was collected by cardiac puncture using a syringe containing heparin (10 U/ml) and used for blood cell count analysis (ABC Vet, Horiba ABX) and preparation of plasma. Plasma was obtained by centrifugation (2000 *× g*, 10 min, 4°C) and stored at -80°C. After blood collection, the mice were perfused with PBS containing 0.5 U/ml heparin and the aortic arch, heart, and liver samples were collected for further analysis. In the western blot analysis of liver samples of both AAV8-Pcsk9-treated atherogenic groups we observed highly efficient depletion of the LDL receptor compared with control animals that did not receive AAV8-Pcsk9 (not shown).

### Plasma cholesterol and triglyceride analysis

The concentration of cholesterol of plasma LDL/VLDL lipoproteins was evaluated with HDL and LDL/VLDL Quantitation Kit (Sigma-Aldrich) according to vendor’s protocol. The level of triglycerides was determined with Liquick-Cor TG kit (Cormay, Poland) according vendor’s instruction.

### Histological examination

The hearts and brachiocephalic arteries (BCAs) collected from the atherosclerotic Nrf2^Cdh5tKO^ and Nrf2^flox/flox^ mice were embedded in OCT compound. Sequential cross sections (8 µm thick) were fixed in formalin, immersed in 60% isopropyl alcohol, and stained with freshly prepared Oil Red O working solution (0.5% Oil Red O in isopropanol was mixed with distilled water in a ratio 3:2) for 5 min. Then the sections were briefly immersed in 60% isopropyl alcohol solution and subsequently examined under a light microscope.

### Isolation and culture of bone marrow-derived macrophages

The healthy 5-month-old Nrf2^Cdh5tKO^ and Nrf2^flox/flox^ mice were euthanized by inhalation of an overdose of isoflurane. The BMDM were obtained according to a protocol described previously (29), with modifications. Briefly, bone marrow cells were collected by flushing the femurs and tibias with sterile physiological saline. The cells were washed with PBS, red blood cells were lysed and the remaining cells were cultured in RPMI medium supplemented with 10% fetal bovine serum (FBS, Biowest), penicillin (100 U/ml) and streptomycin (100 µg/ml; pen-strep, Gibco), and 10 ng/ml of purified human recombinant M-CSF (R&D Systems) for 5 days.

### Isolation of Kupffer cells

The Kupffer cells were isolated from the liver of the same animals, which were used for BMDM isolation. Kupffer cells isolation was performed as previously described (28).

### RT-qPCR

The cells were lysed in Fenozol (A&A Biotechnology). Total RNA was isolated using Total RNA Mini kit (A&A Biotechnology) and then reverse transcribed into cDNA using a High-Capacity cDNA Reverse Transcription Kit (Life Technologies) according to the manufacturer’s instruction. The qPCR reaction was performed with primers targeting Nrf2 exon 3, Nrf2 exon 5 sequence, Nqo1 gene, Gclm gene, Eef2 reference gene (Nrf2_ex3_F: 5’-CAGAGACATTCCCATTTGTAG-3’, Nrf2_ex3_R: 5’-ATTCGGGAATGGAAAATAGC-3’, Nrf2_ex5_F: 5’-CATTCCCGAATTACAGTGTC-3’, Nrf2_ex5_R: 5’-GGAGATCGATGAGTAAAAATGG-3’, Nqo1_F: 5’-GGTTTGGAGTCCCTGCCATT-3’ Nqo1_R: 5’-GTGGATCCCTTGCAGAGAGT-3’, Gclm_F: 5’-TGGAGTTCCCAAATCAGCCC-3’, Gclm_R: 5’-CAACTCCAAGGACGGAGCAT-3’, Eef2_F: 5’-GACATCACCAAGGGTGTGCAG-3’, Eef2_R: 5’-TCAGCACACTGGCATAGAGGC-3’) and *AceQ* qPCR SYBR Green Master Mix (Vazyme) in StepOne Plus Real-Time PCR system (Applied Biosystems). All the procedures were performed according to the manufacturer’s instructions. The level of Nrf2 transcriptional activity (exon 5 deletion) was calculated as a relative expression of Nrf2 exon 5 in comparison to Nrf2 exon 3 expression.

### Preparation of single cells suspension from mouse aortas

The aortic arches with main branches including the perivascular tissue were isolated from atherosclerotic Nrf2^Cdh5tKO^ and Nrf2^flox/flox^ mice (n=3 per group) and control normocholesterolemic (no AAV transduction and standard diet) Nrf2^Cdh5tKO^ and Nrf2^flox/flox^ mice (n=4 per group). The aortas of each group of mice were pooled, cut into small pieces, and digested in 2.5 ml enzyme mix from Multi Tissue Dissociation Kit 2 (Miltenyi Biotec) prepared as described in the protocol provided by the vendor. The samples were placed at 37°C for 30 min with shaking (250 RPM). The suspension was gently pipetted every 15 min. Then the cells were filtered through a 70 μm strainer, centrifuged at 500 × g for 5 min, and resuspended in red blood cells lysis buffer (BioLegend). After 5 min, the cells were centrifuged at 500 × g for 5 min, washed with PBS, and resuspended in 300 µl of PBS containing 2% FBS and 200 ng/ml DAPI. To exclude dead cells and collect only the viable ones, the DAPI-negative cells were sorted with MoFlo XPD (Beckman Coulter) cell sorter.

### Library preparation/data analysis/subclustering/DEG analysis

A total of ~12.000 DAPI-negative cells per sample were loaded on a chromium single cell controller to generate single cell GEMs and then cDNA libraries were prepared using Chromium™ Next GEM Single Cell 3’ GEM, Library & Gel Bead Kit v3.1 (10x Genomics, PN-1000121) following manufacturers protocol. Following QC analysis, cDNA libraries were sequenced on Illumina NovaSeq instrument (paired end, single indexing, 28-8-0-91 cycles) at desired sequencing depth of 50 000 reads/cell. Following sequencing output fastq files were mapped to the mm10 mouse reference genome using 10x Genomics Cell Ranger (cellranger-6.1.2) software. Subsequent downstream analysis was performed in R and R studio softwares (packages: Seurat, Patchwork, Dplyr, Harmony and ggplot2). Any possible cell doublets (nFeature>6000) as well as cells expressing less than 200 genes and more than 15% of mitochondrial genes were excluded from analysis. Cells were clustered using Seurat package (v4.3.0). Results were displayed by UMAP and Heatmaps. Main cell populations were annotated based on the expression of 30 marker genes with highest expression within the cluster. Differentially expressed genes between comparable groups were found using *FindMarkers* function with the parameters test.use=wilcox, min.pct=0.2, tresh.use=0.25, the genes were filtered based on the p value <0.05.

### Kegg pathways analysis

The differentially expressed genes (DEGs) were uploaded to the KEGG Pathways (DAVID; version DAVID 2021, Dec. 2021) (30) to identify biological pathways enriched in specific mononuclear phagocyte subtypes of 1) atherosclerotic Nrf2^flox/flox^ mice in comparison to normocholesterolemic Nrf2^flox/flox^ mice and 2) atherosclerotic Nrf2^Cdh5tKO^ mice in comparison to atherosclerotic Nrf2^flox/flox^ mice. The threshold for the statistical significance was p<0.05. The plots were drawn using the “ggplot2” packages in the R software (version 4.2.1).

### Statistical analysis

Results are presented as mean ± standard deviation (SD) unless stated otherwise. The Student’s *t*-test was used to compare the means of two groups and a value of *p*<0.05 was considered statistically significant. Statistical analyses were performed with GraphPad Prism (GraphPadSoftware Inc., San Diego, CA).

## Results

### Expression of Nrf2 is significantly decreased in macrophages from Nrf2^Cdh5tKO^ mice

**Figure 1:**
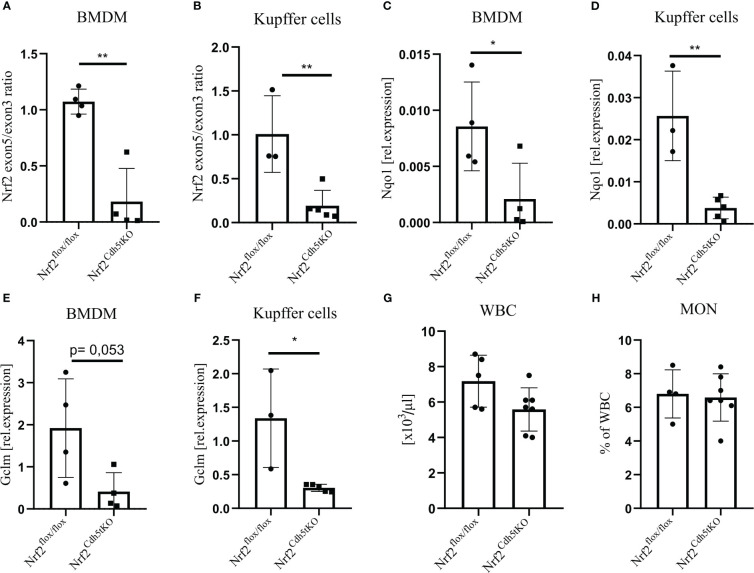
Evaluation of Nrf2 expression in macrophages from Nrf2^Cdh5tKO^ mice. **(A)** The qPCR confirming excision of exon 5 in Nrf2^Cdh5tKO^ BMDM presented as a ratio of exon 5 to exon 3. **(B)** The qPCR confirming the excision of exon 5 Nrf2^Cdh5tKO^ Kupffer cells presented as a ratio of exon 5 to exon 3. **(C)** The qPCR analysis of Nqo1 in BMDM. **(D)** The qPCR analysis of Nqo1 in Kupffer cells. **(E)** The qPCR analysis of Gclm in BMDM. **(F)** The qPCR analysis of Gclm in Kupffer cells. **(G)** The number of WBC in control Nrf2^flox/flox^ and Nrf2^Cdh5tKO^ mice. **(H)** The percentage of blood monocytes in control Nrf2^flox/flox^ and Nrf2^Cdh5tKO^ mice. Results are presented as mean ± SD (*p<0.05, ** p<0.01).

Previous studies investigating the effect of macrophage-specific loss of Nrf2 on atherosclerosis were based on bone marrow transplantation of Nrf2-deficient bone marrow to lethally irradiated Ldlr^−/−^ (25, 31) or ApoE^−/−^ mice (26). Unfortunately, these results did not provide a clear answer on the role of macrophage-specific Nrf2 in atherosclerosis. Here we used animals with transcriptionally inactive Nrf2 in cadherin 5 (Cdh5)-expressing cells (Nrf2^Cdh5tKO^) and the control Nrf2^flox/flox^ mice. In Nrf2^Cdh5tKO^ mice, Cre recombinase excises exon 5 of Nrf2 gene, which is responsible for DNA binding and activation of transcription, making Nrf2 transcriptionally inactive. As a major component of the endothelial cell adherens junction, Cdh5 (VE-cadherin) plays an important role in vascular permeability and angiogenesis (32). Thus, Cdh5-Cre is frequently used for endothelial gene deletion. However, Cdh5-dependent constitutive Cre recombinase expression is present also in a proportion of hematopoietic cells (33–35). To verify whether Nrf2 transcriptional activity is affected in macrophages in our mouse model, we isolated macrophages of two different origins: bone marrow-derived macrophages (BMDM) and Kupffer cells from the liver. For both cell types, we confirmed a decreased level of Nrf2 exon 5 (**Figures 1A, B**) in comparison to Nrf2 exon 3 (the one not affected by Cre recombinase). In support of the decreased Nrf2 activity, we observed lower expression of the two selected Nrf2 target genes: NAD(P)H quinone dehydrogenase 1 (Nqo1) and glutamate-cysteine ligase modifier subunit (Gclm) in BMDM (**Figures 1C, E**, respectively), as well as in Kupffer cells (**Figures 1D, F**, respectively). In addition, the complete blood count analysis showed no difference in white blood cells (WBC, **Figure 1G**) and monocytes (**Figure 1H**) between analyzed healthy mice. It indicates that in steady state conditions decreased transcriptional activity of Nrf2 in hematopoietic and endothelial lineages does not affect formation of WBC and monocytes.

### Pcsk9 overexpression and high fat feeding changes the blood lipids level and induces early stage plaque development in the aortic arch branches of Nrf2^flox/flox^ and Nrf2^Cdh5tKO^ mice

**Figure 2:**
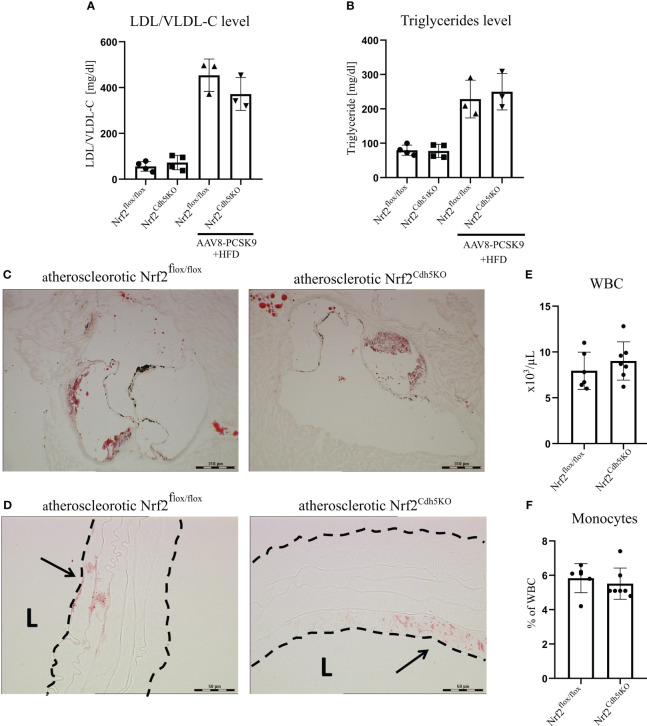
Characterization of atherosclerotic phenotype of Nrf2^flox/flox^ and Nrf2^Cdh5tKO^ mice. **(A)** The level of plasma cholesterol carried by LDL/VLDL lipoproteins in control healthy and atherosclerotic Nrf2^flox/flox^ and Nrf2^Cdh5tKO^ mice. **(B)** The level of plasma triglycerides in control healthy and atherosclerotic Nrf2^flox/flox^ and Nrf2^Cdh5tKO^ mice. **(C)** The staining of lipid deposits within aortic root of Nrf2^flox/flox^ and Nrf2^Cdh5tKO^ mice 21 weeks after AAV8-Pcsk9 injection and HFD. **(D)** The staining of lipid deposits in the brachiocephalic artery of Nrf2^flox/flox^ and Nrf2^Cdh5tKO^ mice 21 weeks after AAV8-Pcsk9 injection and HFD. Lumen of the blood vessel is marked with an ‘L’. The arrows indicate lipid deposits. **(E)** The number of WBC in the blood of Nrf2^flox/flox^ and Nrf2^Cdh5tKO^ mice 21 weeks after AAV8-Pcsk9 injection and HFD. **(F)** The percentage of monocytes in WBC population in the blood of atherosclerotic Nrf2^flox/flox^ and Nrf2^Cdh5tKO^ mice 21 weeks after AAV8-Pcsk9 injection and HFD. Results are presented as mean ± SD.

Our atherosclerosis model was based on intravenous delivery of the AAV8 vector encoding Pcsk9, a protein controlling the expression of LDL receptor in the liver what, in combination with high-fat feeding, induces hypercholesterolemia in mice (36). Two groups of mice were subjected to this treatment: Nrf2^Cdh5tKO^ and Nrf2^flox/flox^. The mouse plasma samples were collected 21 weeks after introducing proatherogenic factors. The analysis revealed increased level of plasma cholesterol carried by LDL/VLDL lipoproteins (**Figure 2A**) and increased level of triglycerides (**Figure 2B**) in atherosclerotic mice when compared to control littermates, with no significant differences between both genotypes. The Oil Red O (ORO) staining of aortic roots cross-sections confirmed the presence of plaques and atherosclerotic phenotype in both groups of mice (**Figure 2C**). The ORO staining of brachiocephalic artery cross-sections showed small lipid deposits within the aortic wall corresponding to an early stage of plaque development in the aortic arch branches of both genotypes (**Figure 2D**). No lipid deposits were detected within the aortic walls of normocholesterolemic Nrf2^flox/flox^ and Nrf2^Cdh5tKO^ mice (data not shown). After 21 weeks of atherosclerosis induction, the complete blood count analysis showed comparable numbers of WBC (**Figure 2E**) and monocytes (**Figure 2F**) in Nrf2^flox/flox^ and Nrf2^Cdh5tKO^ mice.

### Single-cell RNA sequencing identifies several types of mononuclear phagocytes in the aortas of Nrf2^flox/flox^ and Nrf2^Cdh5tKO^ mice

**Figure 3:**
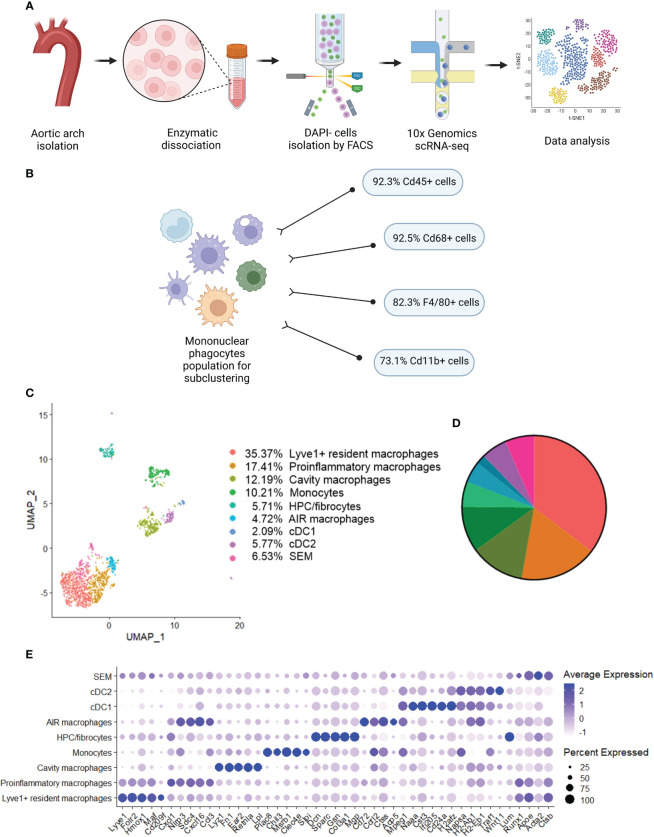
Identification of distinct mononuclear phagocytes in the analyzed fragments of aortas of Nrf2^flox/flox^ and Nrf2^Cdh5tKO^ mice. **(A)** Schematic depiction of the scRNA-seq procedure. **(B)** The percentage of cells expressing classical macrophage markers: *Cd45, Cd68, F4/80, Cd11b* within the whole macrophage population before subclustering. **(C)** UMAP representation of integrated scRNA-seq gene expression data from normocholesterolemic Nrf2^flox/flox^ (n=4) and Nrf2^Cdh5tKO^ (n=4), as well as atherosclerotic Nrf2^flox/flox^ (n=3) and Nrf2^Cdh5tKO^ (n=3) murine aortic arches and branches with identification of the major cell types. **(D)** Proportions of distinct cell types singled out among whole macrophage population. **(E)** Dot plot of average gene expression of the indicated marker transcripts for each cell cluster.

Previous studies using single-cell RNA sequencing (scRNA-seq) highlighted the complexity of macrophage biology and their heterogeneity in human and murine aortas (7, 9). In our study we applied this technology to analyze the transcriptomic changes in different macrophage subpopulations deficient in Nrf2 transcriptional activity in the context of their possible contribution to atherosclerosis development. We collected control and atherosclerotic aortic arches with branches from Nrf2^flox/flox^ and Nrf2^Cdh5tKO^ mice. Then, the pooled fragments of aortas of each group were subjected to scRNA-seq (**Figure 3A**). The unsupervised clustering of 14 125 cells identified, among others, two clusters of macrophages. They were annotated as proinflammatory and tissue-resident based on the expression of *Pf4, Mrc1, C1qa, C1qb, C1qc* genes, and *Lyz1, Rentla, Lyz2, Cd74* genes, respectively. Within the merged (proinflammatory and tissue-resident) macrophage population of 1821 cells, 92.3% were Cd45(*Ptprc*)-positive, 92.5% were Cd68-positive, 82.3% were F4/80(*Adgre1*)-positive, and 73.1% were Cd11b(*Itgam*)-positive (**Figure 3B**). The variation in the content of these immune cell markers suggested that the population is heterogenous and, besides macrophages, consists of other cell types. The subclustering analysis of this heterogenous population, here called mononuclear phagocytes, revealed 9 subpopulations (**Figure 3C**) with a high abundance of lymphatic vessel endothelial receptor-1 (Lyve1+) resident macrophages, proinflammatory macrophages, cavity macrophages and monocytes (**Figure 3D**). The other subtypes - the population of stem, endothelial and mesenchymal (SEM) cells, hematopoietic progenitor cells (HPC)/fibrocytes, aortic intima resident (AIR) macrophages, conventional type 1 dendritic cells (cDC1) and conventional type 2 dendritic cells (cDC2) were less abundant (**Figure 3D**). Lyve1+ resident macrophages were characterized according to previous reports (8, 9, 37) by the expression of *Lyve1, Folr2, Hmox1, Maf*, and *Cd209f*, whereas cavity macrophages (8) by *Lyz1, Fn1, Ear2, Rentla*, and *Lpl* (**Figure 3E**). The expression of *Cxcl1, Nlrp3, Sdc4, Cxcl16*, and *Ccl3* genes characterized both proinflammatory and AIR macrophages (8), but the presence of typical AIR macrophage markers *Cd72, Ccrl2, Ctss, Acp5, Mpeg1* (8) distinguished this population from the proinflammatory subset (**Figure 3E**). Monocyte cluster (8) showed high expression of *Plac8, Chil3, Msrb1, Clec4e, and Slpi* (**Figure 3E**); HPC/fibrocytes (38) were enriched in *Dcn, Sparc, Gsn, Col3a1, Mgp* (**Figure 3E**), while SEM cluster (39) highly expressed *Lum, Runx1, Apoe, Acta2, Ctsb* (**Figure 3E**). For cDC1 (8) and cDC2 (8) we found overlapping expression of classical marker genes with higher expression of *Naaa, Cst3, Ifi205, Cd24a, H2afz* for cDC1 (**Figure 3E**) and *Napsa, H2-ab1, H2-eb1, Traf1, Wnt11* for cDC2 (**Figure 3E**).

**Figure 4:**
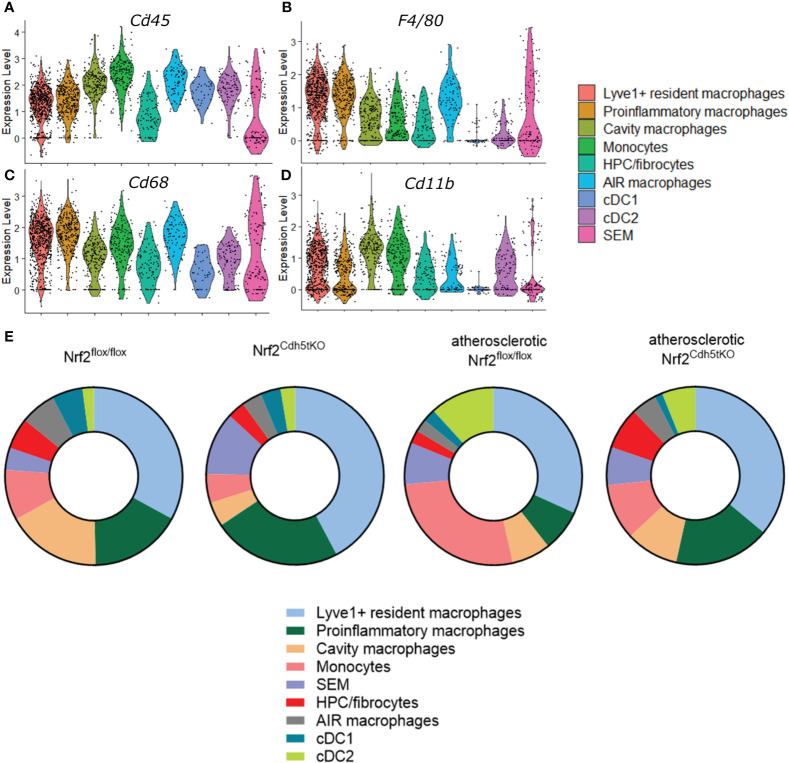
Characteristics of the identified mononuclear phagocyte clusters. The expression of typical macrophage genes **(A)** Cd45, **(B)** F4/80, **(C)** Cd68, **(D)** Cd11b within particular mononuclear phagocyte population shown in the violin plot. **(E)** Proportions of identified cell subsets in the analyzed fragments of aortas of control normocholesterolemic and atherosclerotic Nrf2^flox/flox^ and Nrf2^Cdh5tKO^ mice.

The analysis of classical macrophage markers - *Cd45, Cd68, F4/80, Cd11b* (**Figures 4A–D**, respectively) revealed their lowest expression in HPC/fibrocytes and SEM cells. Moreover, the level of *Cd68, F4/80*, and *Cd11b* (**Figures 4B–D**, respectively) was moderately or strongly decreased in cDC1 and cDC2 cell clusters. Analysis of the abundance of various populations of mononuclear phagocytes in the aortas of healthy and atherosclerotic mice of both genotypes revealed a higher percentage of monocytes (9.18% and 5.25% respectively for normocholesterolemic Nrf2^flox/flox^ and Nrf2^Cdh5tKO^ *vs*. 27.06% and 10.17% respectively for atherosclerotic Nrf2^flox/flox^ and Nrf2^Cdh5tKO^) and cDC2 cells (2.05% and 2.62% respectively for normocholesterolemic Nrf2^flox/flox^ and Nrf2^Cdh5tKO^ *vs*. 11.76% and 6.10% respectively for atherosclerotic Nrf2^flox/flox^ and Nrf2^Cdh5tKO^) in atherogenic conditions (**Figure 4E**). Interestingly, in atherosclerotic Nrf2^Cdh5tKO^ mice a lower proportion of monocytes was associated with a higher proportion of proinflammatory macrophages (17.44% for atherosclerotic Nrf2^Cdh5tKO^ *vs*. 7.65% for atherosclerotic Nrf2^flox/flox^).

### The transcriptome of distinct aortic mononuclear phagocytes is differently affected by decreased Nrf2 transcriptional activity under atherogenic conditions

Next, we analyzed macrophage subtype-specific gene expression using scRNA-seq technology in the aortic arches and branches from control normocholesterolemic and atherosclerotic mice of two genotypes - Nrf2^flox/flox^ and Nrf2^Cdh5tKO^. We focused mostly on three highly abundant clusters that play an important role in plaque development: Lyve1+ resident macrophages, proinflammatory macrophages and monocytes (**Figure 3C**).

#### Lyve1+ resident macrophages

**Figure 5:**
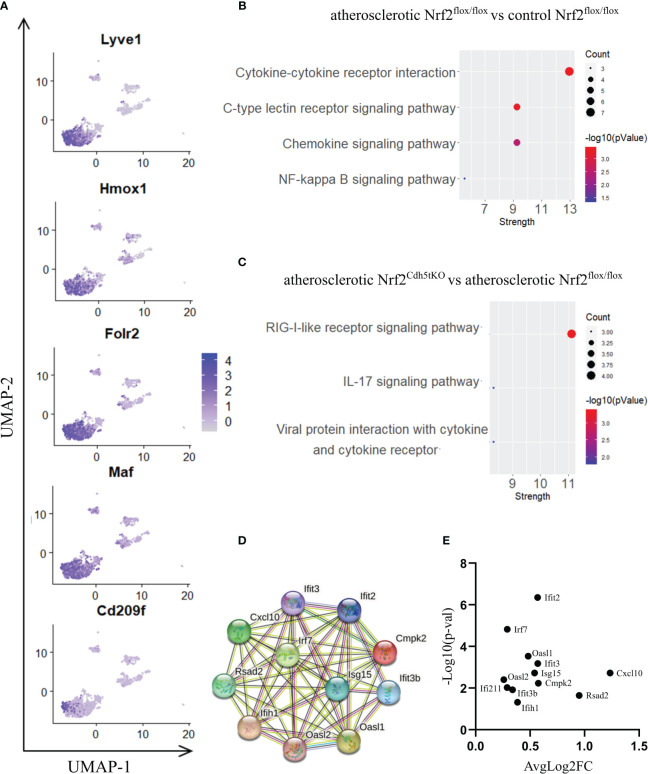
The analysis of transcriptomic changes in Lyve1+ resident macrophages. **(A)** Expression of marker genes identified for Lyve1+ resident macrophages projected onto the UMAP plot. **(B)** KEGG pathway enrichment analysis of upregulated and downregulated genes for atherosclerotic Nrf2^flox/flox^ *vs*. control Nrf2^flox/flox^ and **(C)** atherosclerotic Nrf2^Cdh5tKO^ *vs*. atherosclerotic Nrf2^flox/flox^ Lyve1+ resident macrophages. The x-axis represents the strength (Log10(observed/expected)) of enriched pathways, whereas the color denotes –log10(pValue) and dot size corresponds to gene count in each pathway. **(D)** STRING interaction network - graphical representation of Interferon-simulated genes (ISG) cluster. **(E)** Volcano plot of the top differentially expressed ISG in atherosclerotic Nrf2^Cdh5tKO^ *vs*. atherosclerotic Nrf2^flox/flox^ Lyve1+ resident macrophages.

Lyve1+ resident macrophages are considered to originate from embryonic erythro-myeloid progenitors (40) but, under certain circumstances, circulating monocytes can differentiate into self-maintaining tissue-resident macrophages that resemble their embryonic counterparts. In our analysis, this macrophage subset is characterized by high expression of *Lyve1, Folr2, Hmox1, Maf*, and *Cd209f* (**Figures 3E**, **5A**), and was the most numerous subpopulation in all samples subjected to scRNA-seq (**Figure 4E**). First, we analyzed how atherosclerosis itself affects gene expression in this macrophage subset. In Lyve1+ resident macrophages of atherosclerotic Nrf2^flox/flox^ mice, we identified 16 downregulated and 38 upregulated genes when compared to normocholesterolemic Nrf2^flox/flox^ controls (**Table 1**). The KEGG pathway analysis (**Figure 5B**) showed that many differentially expressed genes (DEGs) were significantly enriched in cytokine-cytokine receptor interaction (*Ccl9, Bmp2, Gdf15, Il1b, Ccl4, Cxcl13, Ccr2*), C-type lectin receptor signaling pathway (*Cd209d, Clec4b1, Cd209b, Il1b, Lsp1*), chemokine signaling pathway (*Lyn, Ccl9, Ccl4, Cxcl13, Ccr2*), NF-kappa B signaling pathway (*Lyn, Il1b, Ccl4*). The majority of the identified DEGs were associated with a pro-inflammatory cell phenotype.

**Table 1:** DEGs in Lyve1+ resident macrophages (atherosclerotic Nrf2^flox/flox^ *vs*. normocholesterolemic Nrf2^flox/flox)^.

Upregulated		
Gene	p-value	avg_log2FC
***Cxcl13***	2,39E-08	0,861436
***Ccr2***	1,47E-07	0,701267
***Arhgdib***	0,032756	0,641391
***Gfra2***	4,55E-07	0,488265
***Btg1***	0,04049	0,472143
***Furin***	0,008615	0,468665
***Cbr2***	0,042708	0,448178
***Tyrobp***	0,021066	0,442919
***Cd209d***	0,002953	0,436895
***Gm42418***	0,039606	0,396878
***Ugdh***	8,43E-11	0,389207
***Pde8a***	0,00899	0,379347
***Mtus2***	1,19E-13	0,377547
***Cd68***	0,023115	0,376554
***F830016B08Rik***	1,32E-13	0,354216
***Fcrls***	0,023415	0,347959
***S100a8***	0,000941	0,336188
***Bmp2***	0,000161	0,330332
***Fstl1***	1,66E-17	0,321947
***Gpr183***	0,008656	0,316971
***Cited2***	0,014093	0,31122
***Ppp1r12a***	0,025934	0,310632
***Smc4***	1,67E-05	0,30858
***Nfkbid***	0,034186	0,307896
***Ccnd1***	1,63E-06	0,301505
***Iigp1***	5,58E-12	0,295462
***Anp32e***	0,001897	0,293936
***Ifi213***	5,80E-12	0,285891
***Apobec1***	5,18E-05	0,285051
***Cd209b***	0,022909	0,28255
***Odc1***	0,002432	0,282423
***Igfbp4***	0,011171	0,277896
***Tsc22d1***	5,64E-05	0,277236
***Clec5a***	6,09E-10	0,276247
***Mfap4***	1,17E-09	0,274002
***Ramp1***	2,78E-11	0,271583
***Myl9***	3,51E-05	0,264222
***Nr4a3***	0,000564	0,251389
Downregulated		
Gene	p-value	avg_log2FC
***S100a4***	0,027411	-0,77728
***Ccl4***	0,002927	-0,74716
***Il1b***	2,66E-14	-0,49265
***Gdf15***	0,047466	-0,45099
***Lyve1***	0,007756	-0,36497
***Ccl9***	0,036021	-0,36179
***Adgre5***	0,046157	-0,33137
***Lyn***	0,015787	-0,30115
***Cd300c2***	0,044796	-0,29036
***Lilra5***	0,008648	-0,2749
***Clec4b1***	3,51E-11	-0,27359
***Marcksl1***	0,036684	-0,2674
***Tuba4a***	1,44E-05	-0,26151
***Fxyd5***	0,042591	-0,25779
***Spic***	4,08E-09	-0,25625
***Lsp1***	0,009876	-0,25385

Next, we investigated the effect of Nrf2 deficiency on the transcriptome of Lyve1+ resident macrophages under atherogenic conditions. The scRNAseq analysis revealed that in this macrophage subset, 17 genes were downregulated and 20 were upregulated in atherosclerotic Nrf2^Cdh5tKO^ *vs*. atherosclerotic Nrf2^flox/flox^ mice (**Table 2**). The DEGs were subjected to KEGG functional enrichment analysis (**Figure 5C**). Among the significantly enriched KEGG pathways, we found retinoic acid-inducible gene I (RIG-I)-like receptor signaling pathway (*Ifih1, Cxcl10, Irf7, Isg15*), IL-17 signaling pathway (*Cxcl10, Ccl7, S100a8*), viral protein interaction with cytokine and cytokine receptor (*Cxcl10, Ccl7, Ccr2*).

**Table 2:** DEGs in atherosclerotic Nrf2^Cdh5tKO^ Lyve1+ resident macrophages (atherosclerotic Nrf2^Cdh5tKO^ *vs*. atherosclerotic Nrf2^flox/flox^).

Upregulated		
Gene	p-value	avg_log2FC
***Cxcl10***	0,001899	1,233588
***Rsad2***	0,022622	0,94986
***Cmpk2***	0,00588	0,574843
***Ifit3***	0,000666	0,569893
***Ifit2***	4,45E-07	0,568756
***Isg15***	0,001899	0,540737
***Oasl1***	0,000293	0,482284
***Ifih1***	0,048154	0,383909
***Pbxip1***	2,95E-09	0,344674
***Ifit3b***	0,012109	0,338988
***Ptgs1***	0,004127	0,320827
***Adgre5***	0,000181	0,318622
***Bcl2a1b***	0,003372	0,317612
***Cd93***	0,001162	0,30791
***Rasgef1b***	0,000174	0,302515
***S100a4***	0,022434	0,299475
***Rgs1***	1,99E-07	0,296326
***Irf7***	1,51E-05	0,290236
***Ifi211***	0,009514	0,288928
***Oasl2***	0,003925	0,258254
Downregulated		
Gene	p-value	avg_log2FC
***Ccl7***	0,026876	-0,63902
***Gm12840***	4,76E-07	-0,61068
***Fcrls***	0,003512	-0,52932
***Arhgdib***	0,019785	-0,50975
***Gfra2***	0,000528	-0,45824
***Ugdh***	3,77E-05	-0,43686
***Slc40a1***	3,25E-07	-0,4262
***S100a8***	1,13E-05	-0,42252
***Mtus2***	5,65E-09	-0,3913
***Tnfaip2***	5,40E-06	-0,35575
***Ccr2***	3,42E-05	-0,31412
***Cd209d***	0,021881	-0,30924
***F830016B08Rik***	0,000383	-0,28949
***Mia***	1,11E-07	-0,28862
***Ramp1***	0,02397	-0,27211
***Gadd45g***	0,003512	-0,27173
***Cd209b***	0,005131	-0,27114

RIG-I not only participates in antiviral signaling pathways, but may also affect non-viral diseases such as atherosclerosis (41, 42). Once activated, RIG-I initiates a signaling cascade that leads to the activation of transcription factors, including interferon regulatory factors (IRFs) and nuclear factor kappa B (NF-κB), which induce the expression of interferon-related genes and pro-inflammatory cytokines (43). In fact, our results confirmed that observation revealing upregulation of interferon-stimulated genes (ISGs, **Figures 5D, E**), *Rsad2, Cxcl10*, *Irf7*, *Ifit2, Ifit3, Ifit3b, Ifih1, Isg15, Oasl1, Oasl2, Cmpk2*, in Nrf2^Cdh5tKO^ Lyve1+ resident macrophages highlighting that over 50% of upregulated genes were associated with downstream IFN-response. Among them, the key transcription factor interferon regulatory factor 7 (*Irf7*) was upregulated. Interestingly, comparison of normocholesterolemic Nrf2^Cdh5tKO^ *vs*. normocholesterolemic Nrf2^flox/flox^ mice revealed increased, among others, expression of *Rsad2* and *Oasl1* (**Table 3**) suggesting that Nrf2-defficient Lyve1+ resident macrophages can be predisposed to develop an interferon-stimulated response under favorable proatherogenic conditions. In terms of atherosclerosis, we also found that the top upregulated in the atherosclerotic Nrf2^Cdh5tKO^ mice *Cxcl10* gene, was involved in all the most enriched KEGG pathways. Its altered expression, together with other chemokine and chemokine receptors such as *Ccl7, Ccr2, Cd97, Rgs1* and *S100a8*, may play a role in leukocyte chemotaxis and migration.

**Table 3:** DEGs in normocholesterolemic Nrf2^Cdh5tKO^ Lyve1+ resident macrophages (normocholesterolemic Nrf2^Cdh5tKO^ *vs*. normocholesterolemic Nrf2^flox/flox)^.

Upregulated		
Gene	p-value	avg_log2FC
***Mucl1***	4,80E-52	0,456824
***Rsad2***	1,47E-09	0,452826
***Myl4***	1,77E-07	0,444174
***Oasl1***	5,31E-06	0,41565
***H2-Eb1***	0,010924	0,387727
***Cd36***	0,00958	0,38566
***Sdc3***	0,019887	0,343128
***Ccr2***	7,64E-18	0,341965
***Cd209f***	4,75E-07	0,336083
***Cd209g***	7,93E-14	0,319793
***H2-DMb1***	0,001735	0,312869
***Cryab***	1,02E-07	0,312213
***Arhgdib***	0,029654	0,281278
***Slc15a3***	0,038431	0,278945
***Gm21188***	7,36E-07	0,260851
Downregulated		
Gene	p-value	avg_log2FC
***S100a4***	0,000526	-0,63943
***Retnla***	0,000711	-0,3919
***Id2***	0,037052	-0,38605
***Atf3***	0,014253	-0,37579
***Apoe***	0,00021	-0,30321
***Ctla2a***	2,93E-35	-0,29131
***Il1b***	0,013429	-0,2505

Among DEGs in atherosclerotic Nrf2^Cdh5tKO^ Lyve1+ resident aortic macrophages (**Table 2**), we also identified genes involved in different aspects of cell death regulation. *Slc40a1*, whose expression may be indirectly regulated by Nrf2 (18), encodes ferroportin and has been implicated in the regulation of iron homeostasis and ferroptotic cell death (44). In addition, we detected the upregulation of *Pbxip1* gene which has been shown to promote apoptosis by inhibiting anti-apoptotic Bcl-2 family members, while on the other hand, the anti-apoptotic *Bcl2a1b* gene was upregulated. We also observed changes in *Gadd45g*, involved in promoting cell death in response to DNA damage and other stress signals (45); *Ifi211* that may inhibit cell growth *via* p53/TP53 and RB1-dependent and independent pathways (46); *Tnfaip2* that may regulate cell death in the context- and cell-dependent manner (47, 48).

#### Proinflammatory macrophages

**Figure 6:**
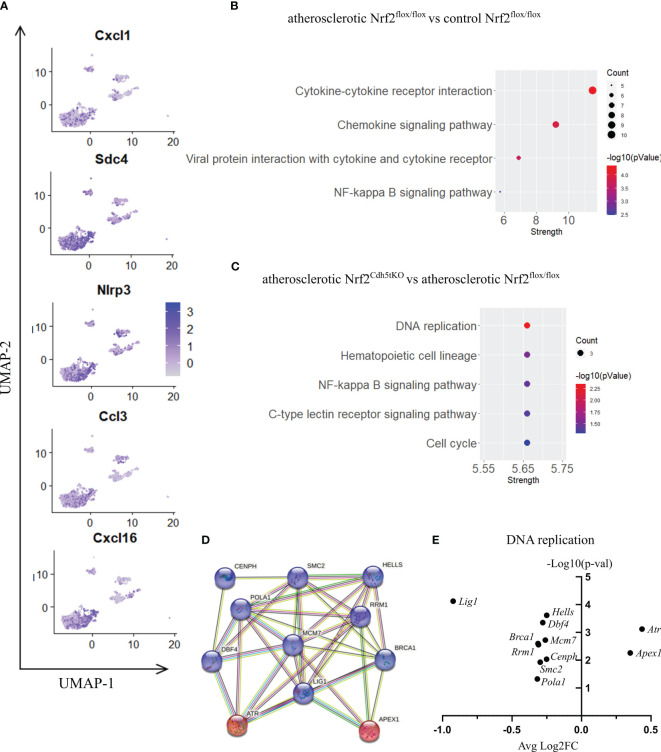
The analysis of transcriptomic changes in proinflammatory macrophages. **(A)** Expression of marker genes identified for proinflammatory macrophages projected onto the UMAP plot. **(B)** KEGG pathway enrichment analysis of upregulated and downregulated genes for atherosclerotic Nrf2^flox/flox^ *vs*. control Nrf2^flox/flox^ and **(C)** atherosclerotic Nrf2^Cdh5tKO^ *vs*. atherosclerotic Nrf2^flox/flox^ proinflammatory macrophages. The x-axis represents the strength (Log10(observed/expected)) of enriched pathways, whereas the color denotes –log10(pValue) and dot size corresponds to gene count in each pathway. **(D)** STRING interaction network - graphical representation of DNA replication and repair genes cluster. The upregulated genes are presented in red color, the downregulated in blue. **(E)** Volcano plot of the top differentially expressed DNA replication and repair genes in atherosclerotic Nrf2^Cdh5tKO^ *vs*. atherosclerotic Nrf2^flox/flox^ proinflammatory macrophages.

Proinflammatory macrophages are the subtype of immune cells mostly derived from monocytes infiltrating the tissues in response to different environmental stimuli. In our scRNAseq analysis, proinflammatory macrophages were the second most abundant cell population within aortic wall, characterized by *Cxcl1, Nlrp3, Sdc4, Cxcl16, Ccl3* marker genes (**Figures 3E**, **6A**). The atherogenic conditions led to the downregulation of 29 and upregulation of 38 genes (**Table 4**) in proinflammatory macrophages of Nrf2^flox/flox^ mice when compared to the normocholesterolemic Nrf2^flox/flox^ controls. Among the KEGG enriched pathways (**Figure 6B**), we identified cytokine-cytokine receptor interaction (*Cxcl10, Cxcl9, Cd4, Cxcl12, Ccl6, Il1b, Il21r, Il2rg, Tnfsf13b, Ccr2*), chemokine signaling pathway (*Lyn, Cxcl10, Hck, Itk, Cxcl9, Cxcl12, Ccl6, Ccr2*), viral protein interaction with cytokine and cytokine receptor (*Cxcl10, Cxcl9, Cxcl12, Ccl6, Il2rg, Ccr2*), NF-kappa B signaling pathway (*Lyn, Cxcl12, Lck, Il1b, Tnfsf13b*). Here, similarly to Lyve1+ resident macrophages, the proatherogenic conditions altered mainly the expression of genes involved in immune responses and proinflammatory cell phenotype.

**Table 4:** DEGs in proinflammatory macrophages (atherosclerotic Nrf2^flox/flox^ *vs*. normocholesterolemic Nrf2^flox/flox)^.

Upregulated		
Gene	p-value	avg_log2FC
***Trbc1***	2,15E-17	1,504752
***H2-M2***	7,87E-14	1,376942
***Il1b***	0,000461	1,193289
***Glipr1***	0,002198	1,0943
***Ms4a7***	0,002158	0,758854
***Tmsb4x***	0,001944	0,746055
***Tyrobp***	3,16E-05	0,663754
***Dusp2***	0,004814	0,653287
***Mylk***	2,71E-05	0,625095
***Hist1h1c***	3,07E-06	0,529187
***Cd24a***	4,03E-32	0,463632
***Dpep2***	0,000217	0,428303
***Tcf7***	1,22E-09	0,39592
***Dusp10***	7,23E-08	0,392107
***Zcwpw1***	0,003704	0,385932
***Clec4d***	7,19E-05	0,381738
***Tnfsf13b***	0,000751	0,368559
***Plk4***	1,29E-16	0,366538
***Rag1***	3,48E-12	0,362176
***Hist1h4d***	0,000195	0,355199
***Jph2***	0,001703	0,348627
***Klra2***	1,12E-05	0,338862
***Brip1***	2,60E-12	0,335571
***Trbc2***	3,43E-05	0,31769
***Cd4***	1,36E-09	0,316175
***Lck***	2,24E-07	0,308246
***Areg***	4,52E-14	0,290733
***Cxcl10***	8,60E-17	0,289706
***Rcan1***	4,46E-07	0,283454
***Itk***	9,96E-18	0,278292
***Clspn***	3,65E-11	0,277299
***Ezh2***	2,23E-09	0,272555
***Slc7a11***	8,49E-06	0,272049
***Dbf4***	1,84E-12	0,262024
***3830403N18Rik***	0,003334	0,256676
***Gimap6***	0,000261	0,250604
***Smc2***	1,58E-11	0,250261
***Pclaf***	5,46E-20	0,250237
Downregulated		
Gene	p-value	avg_log2FC
***Ccl6***	0,003904	-1,37766
***Lyz1***	0,040356	-1,12272
***Mgl2***	0,005566	-1,0127
***Pltp***	0,007962	-0,9206
***Frmd4b***	0,001727	-0,79907
***Cbr2***	0,019255	-0,76134
***Clec10a***	2,96E-06	-0,73313
***Mt1***	0,028923	-0,71533
***Lyn***	0,028313	-0,59088
***Cxcl9***	7,68E-13	-0,56984
***Ciita***	1,60E-05	-0,51057
***Tppp3***	0,008265	-0,49995
***Slamf8***	0,025101	-0,48998
***Cd53***	0,038255	-0,38001
***Hmgn5***	0,003166	-0,37605
***Dcn***	0,037169	-0,35097
***Cxcl12***	0,03321	-0,34359
***Apex1***	0,02609	-0,33454
***Ptgis***	0,000182	-0,32926
***Lrmp***	0,011707	-0,32879
***Ifi209***	0,003534	-0,32394
***Pla2g2d***	0,000148	-0,30379
***Cav2***	0,00013	-0,29992
***Dqx1***	7,97E-35	-0,29513
***Rnasel***	0,012607	-0,29187
***Tsc22d1***	0,032091	-0,28398
***Gpr34***	0,024385	-0,27839
***Myh11***	0,04207	-0,27628
***Gpr183***	0,013806	-0,27444

Nrf2 deficiency additionally modified the transcriptome of these cells. The scRNAseq analysis revealed that 38 genes were downregulated and 15 genes were upregulated in proinflammatory macrophages of atherosclerotic Nrf2^Cdh5tKO^ in comparison to atherosclerotic Nrf2^flox/flox^ (**Table 5**). The KEGG pathway analysis (**Figure 6C**) revealed that the majority of DEGs were associated with DNA replication (*Pola1, Lig1, Mcm7*), hematopoietic cell lineage (*Cd14, Il7r, Cd34*), NF-kappa B signaling pathway (*Lyn, Lck, Cd14*), C-type lectin receptor signaling pathway (*Clec4d, Cd209f, Clec4e*) and cell cycle (*Dbf4, Mcm7, Atr*). Interestingly, comparison of normocholesterolemic Nrf2^Cdh5tKO^ *vs*. normocholesterolemic Nrf2^flox/flox^ mice revealed that the top upregulated gene in proinflammatory macrophages was *Il1b* (**Table 6**). This, together with other DEGs involved in cytokine-cytokine receptor interaction, antigen processing and presentation, cell adhesion molecules, or toll-like receptor pathway, may suggest a more inflammatory phenotype of this Nrf2-deficient macrophage subtype already in steady state conditions.

**Table 5:** DEGs in atherosclerotic Nrf2^Cdh5tKO^ proinflammatory macrophages (atherosclerotic Nrf2^Cdh5tKO^ *vs*. atherosclerotic Nrf2^flox/flox^).

Upregulated		
Gene	p-value	avg_log2FC
***Cd209f***	9,05E-05	1,042335
***Frmd4b***	0,016363	0,828339
***Lyn***	0,02315	0,670389
***Slc6a6***	0,042782	0,476034
***Atr***	0,000761	0,43571
***Mctp1***	0,0184	0,399953
***Arrdc3***	1,52E-06	0,366464
***Apex1***	0,00551	0,350366
***Ifi209***	0,006861	0,342648
***Gm21188***	0,001551	0,313762
***C3***	0,035912	0,297601
***Uap1***	0,016363	0,297434
***Fbxo30***	0,038538	0,276554
***Hmgn5***	0,011867	0,272808
***Il7r***	0,014231	0,256876
Downregulated		
Gene	p-value	avg_log2FC
***Fabp5***	0,010931	-1,9532
***Trbc1***	0,024954	-1,47603
***Mmp12***	0,009443	-1,34337
***Glipr1***	0,005759	-1,07391
***Lig1***	7,56E-05	-0,92399
***S100a4***	0,00527	-0,84746
***Ccr2***	0,032259	-0,70196
***Satb1***	0,002522	-0,67599
***Cd14***	0,02315	-0,64989
***Gpnmb***	0,012874	-0,6213
***Clec4e***	0,002522	-0,61655
***Ccr9***	0,000102	-0,60723
***Ms4a7***	0,016363	-0,60247
***Clec4d***	0,045826	-0,53573
***Tyrobp***	0,012874	-0,44672
***Il1rn***	0,022291	-0,42433
***Dusp10***	0,042782	-0,40252
***Ptprcap***	0,000154	-0,38418
***Cd34***	0,027888	-0,36934
***Cp***	0,042782	-0,35777
***AA467197***	0,003045	-0,353
***Jph2***	0,026877	-0,33817
***Areg***	0,002404	-0,33282
***Pola1***	0,047415	-0,31824
***Brca1***	0,002522	-0,31297
***Rrm1***	0,002773	-0,30989
***Cd247***	0,000194	-0,29907
***Smc2***	0,011867	-0,29682
***Dbf4***	0,000446	-0,2788
***Prrx1***	0,010931	-0,27569
***Lck***	0,004801	-0,27034
***Rmnd5a***	0,041325	-0,26977
***Ctla2a***	0,000145	-0,26973
***Adgre4***	2,33E-06	-0,26094
***Gem***	0,002644	-0,26062
***Mcm7***	0,001888	-0,26053
***Cenph***	0,009222	-0,25088
***Hells***	0,000243	-0,25018

**Table 6:** DEGs in normocholesterolemic Nrf2^Cdh5tKO^ proinflammatory macrophages (normocholesterolemic Nrf2^Cdh5tKO^ *vs*. normocholesterolemic Nrf2^flox/flox)^.

Upregulated		
Gene	p-value	avg_log2FC
***Il1b***	0,027338	0,912593
***Pou2f2***	0,000477	0,515043
***Errfi1***	0,012599	0,397658
***Entpd1***	0,00028	0,394454
***Cd300a***	0,011064	0,360637
***Tmsb4x***	6,67E-07	0,349386
***Cd209f***	7,32E-10	0,347549
***Bmp2***	0,026157	0,341992
***Lst1***	0,007295	0,338405
***Basp1***	0,011608	0,331997
***Tyrobp***	3,07E-05	0,329781
***Cotl1***	0,036986	0,326228
***Pde4b***	0,016117	0,308501
***Zmynd15***	5,06E-06	0,289084
***Runx1***	0,012652	0,282018
***Mctp1***	0,005341	0,279978
***Tgfbi***	0,02097	0,277903
***Myl6***	0,036142	0,272899
***Ccr1***	1,50E-06	0,272622
***Ncf4***	0,002907	0,265326
***Actc1***	1,06E-07	0,264654
***Ranbp1***	0,042688	0,256888
Downregulated		
Gene	p-value	avg_log2FC
***Retnla***	0,048006	-2,55668
***Gdf15***	0,001614	-0,78194
***S100a4***	0,035294	-0,74965
***Lyz1***	1,64E-05	-0,68443
***Mgl2***	2,43E-05	-0,58338
***Cxcl9***	4,65E-42	-0,54531
***Vcam1***	0,000307	-0,53983
***Ccl3***	0,044231	-0,52939
***Pepd***	2,60E-05	-0,46906
***H2-Ab1***	0,007047	-0,4344
***H2-Aa***	0,04455	-0,36261
***H2-Eb1***	0,027323	-0,32848
***Cd74***	0,00508	-0,31822
***Clec10a***	0,006858	-0,31712
***Dqx1***	1,71E-39	-0,30632
***Plbd1***	0,021344	-0,28927
***Lcp2***	0,00315	-0,25308
***Pcna***	0,000764	-0,25137

Moreover, in atherogenic conditions, several DEGs in proinflammatory macrophages of Nrf2^Cdh5tKO^ *vs*. Nrf2^flox/flox^ mice (**Table 5**) were identified as hub genes associated with DNA replication and maintenance (downregulation of *Brca1, Smc2, Cenph, Lig1, Mcm7, Rrm1, Dbf4, Pola1, Hells* and upregulation of *Atr, Apex1*, **Figures 6D, E**). As the *Atr* and *Apex1* genes are the main sensors of oxidative stress-induced DNA damage, we checked for any other genes associated with increased oxidative stress in Nrf2^Cdh5tKO^ proinflammatory macrophages. Among these, we observed upregulation of *Lyn*, which is involved in response to DNA damage/redox imbalance (49); *Fbxo3*, which is a part of SCF complex and may be involved in DNA damage response (50); *Hmgn5*, which may regulate histone modification, DNA replication, repair, and gene transcription through binding to chromatin regulators (51). Among the downregulated DEGs, we found *Dbf4* gene which decreases S-phase checkpoint signaling and maintains DNA replication and cell cycle progression induced by DNA damage (52), DNA damage-induced *Areg* (53), *Satb1* that is involved in DNA repair (54), and *Gem* which regulates chromatin remodeling and DNA replication (51).

Some DEGs were associated with the C-type lectin receptor (CLR) signaling pathway. CLRs are widely expressed by myeloid cells. The expression of macrophage-specific members of CLR family were decreased in Nrf2^Cdh5tKO^ proinflammatory macrophages. Among them, we identified *Clec4e* (macrophage inducible C-type lectin; Mincle) and *Clec4d* (macrophage C-type lectin; MCL). The first one is activated by dead cells/necrosis and both of them are able to sense damage-associated molecular patterns (55).

Within the Src family kinases, *Lck* was downregulated, the level of *Ptprcap*, that stabilizes the association of CD45 with *Lck* (56), was decreased, similarly to *Tyrobp* (*Dap12*) that may be phosphorylated by Src kinases. On the other hand, the other Src family proteins, *Cd14* and *Lyn* were upregulated.

#### Monocytes

**Figure 7:**
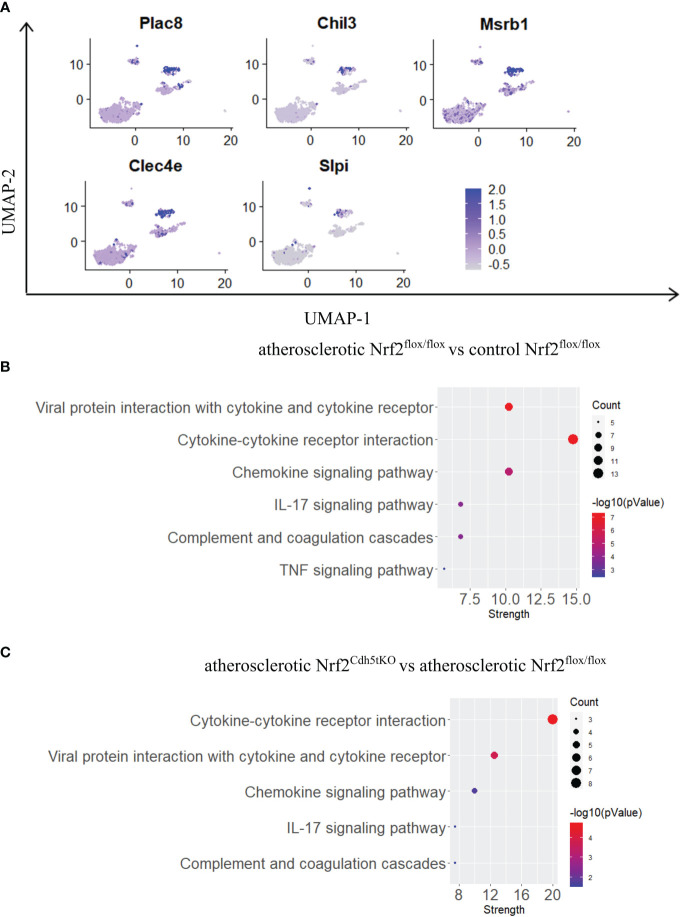
The analysis of transcriptomic changes in monocytes. **(A)** Expression of marker genes identified for monocytes projected onto the UMAP plot. **(B)** KEGG pathway enrichment analysis of upregulated and downregulated genes for atherosclerotic Nrf2^flox/flox^ *vs*. control Nrf2^flox/flox^ and **(C)** atherosclerotic Nrf2^Cdh5tKO^ *vs*. atherosclerotic Nrf2^flox/flox^ monocytes. The x-axis represents the strength (Log10(observed/expected)) of enriched pathways, whereas the color denotes –log10(pValue) and dot size corresponds to gene count in each pathway.

Monocytes possess the ability to infiltrate the tissues and mature into macrophages. In our scRNAseq analysis they were characterized by high expression of *Plac8, Chil3, Msrb1, Clec4e, Slpi* (**Figures 3E**, **7A**) and were one of the most abundant mononuclear phagocyte subset. The monocytes of Nrf2^flox/flox^ mice were strongly affected by the atherogenic conditions what was reflected by the number of DEGs – 146 genes (57 down- and 89 upregulated *vs*. normocholesterolemic Nrf2^flox/flox^ mice; **Table 7**). The KEGG pathway analysis (**Figure 7B**) revealed that many DEGs were related to inflammatory responses: viral protein interaction with cytokine and cytokine receptor (*Ccr1, Il10, Il6, Ccl7, Ccl5, Ccl2, Il2rg, Cxcl3, Pf4*), cytokine-cytokine receptor interaction (*Ccr1, Il10, Il1r2, Inhba, Cxcl3, Il2rg, Cxcl16, Il6, Ccl7, Ccl5, Ccl2, Il7r, Pf4*), chemokine signaling pathway (*Ccr1, Grk3, Gngt2, Ccl7, Ccl5, Ccl2, Cxcl3, Cxcl16, Pf4*), IL-17 signaling pathway (*Il6, Ccl7, Ccl2, Cxcl3, S100a9, S100a8*), complement and coagulation cascades (*C1qb, C4b, C1qa, Serpinb2, C3ar1, C1qc*), genes associated with TNF signaling pathway (*Il6, Ccl5, Ccl2, Cxcl3, Creb5*).

**Table 7:** DEGs in monocytes (atherosclerotic Nrf2^flox/flox^ *vs*. normocholesterolemic Nrf2^flox/flox)^.

Upregulated		
Gene	p-value	avg_log2FC
***S100a9***	4,22E-05	2,244782
***Basp1***	2,38E-10	1,60785
***Retnlg***	9,22E-21	1,577205
***S100a8***	0,004065	1,510919
***Ccl2***	0,000946	1,498106
***Ccl7***	2,41E-09	1,288358
***Spp1***	0,036512	1,215382
***Il1r2***	4,72E-11	1,140975
***Inhba***	1,46E-25	1,117781
***Vegfa***	0,00013	1,082856
***Cxcl3***	3,60E-20	1,080253
***Prg4***	2,20E-25	1,078775
***Rgs1***	0,000105	1,061712
***C5ar1***	0,015234	1,044396
***Cstb***	0,035832	1,023867
***Lilr4b***	0,000666	0,877581
***Sdc4***	3,79E-21	0,873767
***Pf4***	1,58E-07	0,86228
***Lgmn***	0,046372	0,836777
***Mxd1***	1,34E-07	0,829167
***Id2***	0,000335	0,779114
***Dab2***	1,11E-17	0,728129
***Il1b***	0,018683	0,709909
***Clec4d***	0,044953	0,702688
***Ptafr***	0,006671	0,679941
***C3ar1***	7,69E-20	0,654895
***Entpd1***	0,001438	0,645294
***C1qb***	6,42E-10	0,625359
***Marcksl1***	0,042589	0,599681
***Ecm1***	2,83E-05	0,565733
***Runx3***	0,005149	0,558581
***Ms4a7***	1,75E-10	0,555418
***Eps8***	6,21E-05	0,555199
***Nlrp3***	0,040919	0,552225
***Fabp4***	0,047085	0,532537
***Rab11fip1***	0,00121	0,516488
***Rab20***	0,000329	0,45967
***Capg***	0,010068	0,4592
***Plin2***	0,030932	0,443532
***Cxcl16***	0,000537	0,442237
***Slc7a11***	0,048725	0,4358
***Ptgs2***	0,047623	0,435474
***Olr1***	0,002084	0,432372
***Clec4n***	2,21E-06	0,428953
***Dok2***	3,26E-13	0,425217
***Fcgr3***	0,021193	0,422065
***C1qc***	2,77E-16	0,41703
***Adam8***	0,02293	0,404946
***Cd93***	0,024288	0,398992
***Furin***	0,000996	0,395016
***Il6***	4,87E-06	0,391441
***Gpr65***	0,005499	0,38869
***C1qa***	3,74E-08	0,386056
***Pdpn***	8,19E-27	0,382998
***Serpinb2***	0,00494	0,367
***Acod1***	4,72E-16	0,366281
***Odc1***	0,006713	0,363343
***Rab7b***	7,32E-13	0,352893
***Hspa1a***	0,028078	0,352667
***Ankrd33b***	6,81E-17	0,350538
***Trf***	0,000773	0,349309
***Creb5***	1,56E-18	0,343503
***Fam46c***	0,001612	0,34167
***Il7r***	1,80E-09	0,340033
***Ass1***	5,99E-06	0,337812
***Efhd2***	0,036588	0,33655
***Clec5a***	6,12E-16	0,336504
***Rgcc***	0,013707	0,327872
***Ccr1***	4,96E-05	0,324188
***Maf***	0,000167	0,321496
***Ran***	0,015117	0,317006
***C4b***	6,74E-16	0,314411
***Tmem37***	1,78E-21	0,314265
***Nrp2***	2,18E-18	0,311033
***Plekho1***	0,007307	0,307844
***Arg2***	2,74E-08	0,303814
***Glrx***	3,35E-06	0,302841
***Bcl2a1b***	0,012414	0,299724
***Fcgr1***	0,000566	0,299692
***Lacc1***	3,32E-09	0,275824
***Cenpa***	8,29E-14	0,274966
***Cysltr1***	2,91E-14	0,273945
***Tsc22d1***	4,82E-07	0,26936
***Ifit3***	2,14E-07	0,267745
***Bcl2a1d***	0,027019	0,26495
***Mrc1***	1,16E-19	0,264818
***Stab1***	7,27E-19	0,256998
***Cmah***	6,67E-09	0,254944
***Cd86***	0,007618	0,25333
Downregulated		
Gene	p-value	avg_log2FC
***Hes1***	5,73E-08	-1,18421
***Gngt2***	0,00481	-1,02475
***Pou2f2***	0,000119	-0,73768
***Itgal***	0,010299	-0,71632
***Fam107b***	0,000992	-0,62345
***Plac8***	0,009667	-0,59459
***H2-Eb1***	0,010857	-0,58955
***Adgre5***	0,003784	-0,5846
***Spn***	0,02998	-0,58211
***Samhd1***	0,001794	-0,58062
***Il2rg***	0,002521	-0,57655
***Napsa***	0,002012	-0,57128
***Gm9733***	0,00556	-0,56823
***Samsn1***	0,011576	-0,55471
***Ly6i***	1,49E-08	-0,53254
***Ikzf1***	0,000899	-0,50793
***Ceacam1***	0,00772	-0,50464
***Nabp1***	0,000779	-0,49656
***Gsr***	0,00512	-0,49127
***Ptprc***	0,02186	-0,47549
***Coro1a***	0,000812	-0,46232
***C3***	0,01185	-0,43997
***Nfam1***	0,006526	-0,43844
***Stk10***	0,013361	-0,43065
***Ppp1r12a***	0,014837	-0,43023
***Ets1***	6,73E-06	-0,42168
***Grk3***	0,000688	-0,40919
***Bin2***	0,006256	-0,40871
***Ifitm6***	0,036091	-0,40527
***Ear2***	0,001697	-0,3956
***Fgr***	0,036856	-0,38661
***Adgre4***	0,001166	-0,3842
***Cytip***	0,009957	-0,38144
***Tppp3***	0,004165	-0,37381
***Il10ra***	0,017348	-0,37184
***Spi1***	0,039732	-0,3638
***Saa3***	1,04E-17	-0,3629
***H2-Aa***	0,006533	-0,35594
***Lst1***	0,032867	-0,35426
***Ptpn6***	0,038215	-0,35237
***Gm19951***	3,45E-08	-0,35106
***Cbfa2t3***	0,000225	-0,34962
***Ccl5***	1,84E-13	-0,32136
***H2-Ab1***	0,025519	-0,32122
***Limd2***	0,025979	-0,31346
***Il10***	1,04E-17	-0,30918
***Msrb1***	0,032869	-0,30777
***Itgb7***	0,0182	-0,30705
***Adssl1***	0,033508	-0,30264
***Mfap5***	0,013216	-0,29002
***Icam2***	1,22E-05	-0,28042
***Trbc2***	2,74E-11	-0,27078
***Igfbp5***	2,61E-06	-0,2638
***Tyrobp***	0,001737	-0,26266
***Ptpn22***	1,85E-05	-0,26183
***Cd300e***	4,41E-09	-0,25778
***Slpi***	0,002152	-0,25694

In monocytes of atherosclerotic Nrf2^Cdh5tKO^ mice, we found 19 downregulated and 24 upregulated genes when compared to atherosclerotic Nrf2^flox/flox^ (**Table 8**). The KEGG pathway analysis (**Figure 7C**) uncovered 5 enriched pathways. Among these, we found cytokine-cytokine receptor interaction (*Ccr1, Il6, Il1rn, Ccl7, Gdf15, Il1r2, Ccl3, Cxcl13*), viral protein interaction with cytokine and cytokine receptor (*Ccr1, Il6, Ccl7, Ccl3, Cxcl13*), chemokine signaling pathway (*Ccr1, Ccl7, Ccl3, Cxcl13)*, IL-17 signaling pathway (*Il6, Ccl7, S100a9*), complement and coagulation cascades (*C4b, Serpinb2, Vsig4*). The last pathway was the only one to be enriched when normocholesterolemic Nrf2^Cdh5tKO^ and Nrf2^flox/flox^ mice were compared (**Table 9**).

**Table 8:** DEGs in atherosclerotic Nrf2^Cdh5tKO^ monocytes (atherosclerotic Nrf2^Cdh5tKO^ *vs*. atherosclerotic Nrf2^flox/flox^).

Upregulated		
Gene	p-value	avg_log2FC
***Mgl2***	0,000151	1,111916
***Cxcl13***	2,33E-09	1,084349
***Slpi***	0,022409	1,072164
***Mmp12***	0,015239	0,59813
***Ifitm6***	0,044762	0,558191
***AA467197***	0,000115	0,49493
***Hbb-bt***	0,000493	0,443647
***Gm12840***	0,011678	0,438294
***Icam2***	0,001169	0,425176
***Ccl7***	0,003695	0,411656
***Il1rn***	0,048982	0,400177
***Ednrb***	2,25E-08	0,392623
***Timp3***	0,00014	0,368488
***Saa3***	0,000654	0,363723
***Ccr1***	0,039922	0,33807
***Vsig4***	6,93E-06	0,33687
***Gm19951***	2,56E-07	0,328188
***Padi4***	0,001794	0,311341
***Fmo2***	0,000111	0,299015
***Lum***	4,69E-07	0,291819
***Socs2***	0,000256	0,269916
***C4b***	7,76E-05	0,261613
***Serpinb2***	0,003269	0,251719
***Trem3***	0,00595	0,250948
Downregulated		
Gene	p-value	avg_log2FC
***S100a9***	9,80E-07	-2,71359
***Il1r2***	0,000157	-1,04943
***Gdf15***	0,00317	-1,04697
***Ccl3***	0,002041	-0,85663
***Il6***	0,00317	-0,55114
***Lair1***	0,019751	-0,46441
***Sdc4***	0,000229	-0,43374
***Olr1***	0,000427	-0,42507
***2010015M23Rik***	7,77E-08	-0,36718
***Fbxo32***	0,00668	-0,35142
***Maf***	0,003927	-0,33295
***Gm14548***	0,00263	-0,32188
***Dab2***	0,003695	-0,26731
***H2-DMa***	0,031563	-0,26226
***Mrc1***	2,10E-06	-0,26101
***Cenpa***	0,018293	-0,25761
***Gm26870***	7,77E-08	-0,25656
***P2ry6***	0,02798	-0,25297
***Basp1***	0,022976	-0,2505

**Table 9:** DEGs in normocholesterolemic Nrf2^Cdh5tKO^ monocytes (normocholesterolemic Nrf2^Cdh5tKO^ *vs*. normocholesterolemic Nrf2^flox/flox)^.

Upregulated		
Gene	p-value	avg_log2FC
***Thbs1***	0,000882	0,698712
***F13a1***	0,008909	0,637967
***Lbr***	0,01143	0,459449
***Clec4d***	0,047093	0,429932
***Slfn2***	0,032847	0,421368
***Evi2a***	0,018258	0,41203
***Gm26778***	0,030337	0,407959
***Mxd1***	0,007663	0,406864
***Gadd45a***	0,001254	0,394997
***Naaa***	0,015887	0,393913
***Sell***	5,54E-05	0,372804
***Dab2***	0,018307	0,353464
***St8sia4***	0,000414	0,347379
***Ifi211***	0,004227	0,344962
***Mx1***	6,52E-06	0,327849
***Nfkbie***	0,014707	0,315773
***Ifit3***	0,048771	0,313544
***Clec4a2***	0,038668	0,30933
***Eps8***	0,006948	0,308254
***Ciita***	1,21E-06	0,303733
***C5ar1***	2,86E-05	0,297725
***Rnasel***	2,27E-05	0,294714
***Pcdh7***	1,70E-13	0,283886
***Lif***	1,66E-16	0,263507
***Tnip3***	0,034321	0,263488
***Sla***	0,04905	0,262487
Downregulated		
Gene	p-value	avg_log2FC
***Hes1***	0,004695	-1,2028
***C1qa***	0,000305	-0,82092
***S100a8***	0,001518	-0,80354
***C1qc***	1,44E-05	-0,78584
***Prg4***	6,03E-06	-0,64874
***S100a9***	0,003547	-0,61631
***Serpinb2***	0,033818	-0,60127
***Rgs1***	9,27E-06	-0,59562
***Icam2***	5,10E-09	-0,5471
***Cd36***	0,041232	-0,54095
***Hmgn5***	2,92E-06	-0,38335
***Ly6i***	0,025161	-0,38235
***Il10***	1,34E-10	-0,36946
***Unc119***	0,049872	-0,35086
***Hilpda***	7,20E-07	-0,3472
***Ccl5***	5,46E-19	-0,33329
***Ranbp1***	0,029044	-0,33061
***Tuba4a***	0,023782	-0,30078
***Acp5***	0,023156	-0,29388
***Saa3***	4,83E-13	-0,29215
***Trbc2***	1,87E-10	-0,28844
***Gm20186***	1,42E-06	-0,26269

The transcriptome of monocytes from atherosclerotic Nrf2^Cdh5tKO^ mice (**Table 8**) was strongly changed in terms of the expression of genes encoding chemokines, cytokines, and their receptors. Besides the genes associated with cytokine-cytokine receptor interaction (downregulated *Il6, Gdf15, Il1r2, Ccl3* and upregulated *Ccr1, Il1rn, Ccl7, Cxcl13*), in atherosclerotic Nrf2^Cdh5tKO^ monocytes we also identified upregulation of *Ednrb*, which is a receptor for endothelin and angiotensin (57). Interestingly, in monocyte DEGs between atherosclerotic Nrf2^Cdh5tKO^ and Nrf2^flox/flox^ mice, we also found several protease inhibitors, such as *Timp3, Serpinb2, and Slpi*, that may change the activity of peptidases and contribute to vascular remodeling.

## Discussion

Macrophages drive atherosclerosis at all stages of plaque development. It was previously shown that local tissue environment shapes the transcriptome and identity of macrophages and various macrophage types may differentially respond to the same stimuli (58). One of the stimuli acting in atherosclerotic plaques is oxidative stress, which has been implicated in the disease progression from the early fatty streak lesions to advanced atherosclerotic plaques (22). The accumulation of reactive oxygen species within the cells can affect different signaling pathways regulating cell cycle, migration and survival leading to cellular dysfunction and even cell death (59).

In this study, we analyzed the transcriptomic changes caused by atherogenic conditions in different populations of murine aortic mononuclear phagocytes deficient in Nrf2 transcriptional activity. In physiological conditions this redox-activated transcription factor provides protection *via* activation of expression of a number of genes mediating antioxidant response (22). However, Nrf2 effects in atherosclerosis seem to be more complex and currently not completely understood. Here, we analyzed the data obtained from mice with transcriptionally inactive Nrf2 in Cdh5-expressing cells and their progeny. The prevailing scientific consensus is that hematopoiesis, the process of blood cell formation, primarily occurs through two distinct mechanisms during development: yolk-sac hematopoiesis and hemogenic endothelium-derived hematopoiesis. The exact contribution of these sources to adult hematopoiesis is still a subject of active research and ongoing debate (40, 60). Nevertheless, according to the literature data even 50-96% of CD45+ adult bone marrow–derived cells may be affected by Cdh5-dependent Cre recombinase active during embryonic development (34, 35). Therefore, the Cre-driven mutation is usually present not only in endothelial cells, but also in a number of leukocytes. In our mouse model, macrophages of two different origins – BMDM and Kupffer cells - were characterized by significantly decreased level of Nrf2 exon 5 responsible for the transcriptional Nrf2 activity.

Atherosclerosis was induced *via* AAV8-mediated Pcsk9 overexpression and a high-fat diet (36). This approach successfully elevated the cholesterol level in the LDL/VLDL plasma lipoproteins, as well as the level of triglycerides in the mouse plasma in the range previously described for similar proatherogenic approaches (61–63). In the fragments of the aorta (aortic arch and branches) used for scRNAseq procedure, we observed lipids accumulation within the first layers of intima and media corresponding to fatty streaks, which represent an early stage of atherosclerotic plaque formation. In such early stage lesions, macrophage infiltration, initiation of inflammation and chemoattraction of other cell types is usually observed.

Nowadays, mainly with the use of scRNA-seq technology, several macrophage subtypes were identified in mouse and human atherosclerotic plaques, each with distinct functions and properties (7–9, 37, 38). It is well-recognized that monocyte-derived inflammatory macrophages are not the only players in atherosclerotic plaque development. Thus, we performed subclustering analysis to visualize the macrophage heterogeneity and macrophage subtype-specific effects of decreased Nrf2 transcriptional activity. That strategy uncovered 9 subpopulations of mononuclear phagocytes, in majority comprising macrophages of different origin, associated with different layers of aortic wall or peritoneal cavity. Each subpopulation was characterized by the typical markers described in previous reports (8, 9, 37, 38).

We selected three most abundant subtypes, namely Lyve1+ resident macrophages, proinflammatory macrophages and monocytes, for in-depth transcriptomic analysis in proatherogenic conditions. Tissue-resident macrophages maintain tissue homeostasis, remove pathogens and abnormal (apoptotic or senescent) cells, participate in tissue repair and regeneration (60). In the aortic wall, the tissue-resident macrophages are present mainly in adventitia in steady-state conditions, whereas in inflammation they can migrate towards the media and intima layer (64). The proinflammatory macrophages release several proinflammatory cytokines and ingest modified lipids what results in foam cells formation (64). Monocytes are blood-circulating cells that play an important role in atherosclerosis as protagonists of plaque development. When the lining of the artery is damaged, monocytes are intensively recruited to the site of injury.

The comparison between atherosclerotic Nrf2^flox/flox^ and control normocholesterolemic Nrf2^flox/flox^ mice revealed that each analyzed mononuclear phagocytes subset contained DEGs encoding cytokines, chemokines and their receptors involved in immune responses. This confirms that inflammation is an important component in the pathogenesis of atherosclerosis already in the early stage lesions (4, 5). In our analysis, the highest number of DEGs induced by atherogenic conditions was identified in the monocyte subset. Interestingly, among monocyte DEGs between atherosclerotic Nrf2^flox/flox^ and control normocholesterolemic Nrf2^flox/flox^ mice we found upregulation of NOD-like receptor pyrin domain-containing protein 3 (*Nlrp3*) and interleukin 1β (*Il1b*), two components of Nlrp3 inflammasome activation, which normally leads to Il-1β release. Inhibition of this cytokine production is currently of particular interest for the secondary prevention of atherosclerotic events (65).

Next, we investigated the effect of decreased Nrf2 transcriptional activity on the transcriptomic changes in monocytes and selected macrophage subsets in atherosclerotic mice. Previous *in vitro* studies indicated that Nrf2 activity may promote the anti-inflammatory phenotype of macrophages (66, 67), but the effect of Nrf2 on tissue-resident macrophages is still poorly investigated. It was shown that in alveolar macrophages Nrf2 increases phagocytic ability (68), macrophage-driven efferocytosis and apoptotic neutrophil clearance (69), and may protect from ferroptosis in sepsis-induced acute lung injury (20). Other studies performed on dermal resident macrophages showed the involvement of Nrf2 in IL-23–IL-17A–TRPV1 axis and pain perception (70) or wound repair (71). In our study, the RIG-I like pathway was the top altered pathway in atherosclerotic Nrf2^Cdh5tKO^ Lyve1+ resident macrophages. RIG-I may activate IRFs and NF-κB, and then induce the expression of antiviral genes, such as type I interferons and pro-inflammatory cytokines (43). The latest research shows that RIG-I-like pathway is not only triggered by viral nucleic acids, but also by damage-associated molecular patterns (72). It was suggested that Nrf2 can interact with RIG-I-like pathway and modulate its function. Specifically, Nrf2 has been shown to regulate the expression of genes involved in the antiviral response, including interferon-stimulated genes (ISGs) that are downstream effectors of RIG-I-like signaling (41). Interestingly, in Lyve1+ resident macrophages of atherosclerotic Nrf2^Cdh5tKO^ *vs.* atherosclerotic Nrf2^flox/flox^, we also observed upregulation of *Bcl2a1b*, an IFN-dependent master switch for the function of Cd11b+ cells exerting anti-apoptotic and pro-survival effects (42). In addition, in Lyve1+ resident macrophages from atherosclerotic Nrf2^Cdh5tKO^ mice we observed downregulation of ferroportin gene *Slc40a1*. This is in accordance with previous reports describing involvement of Nrf2 in regulation of this gene expression, as well as in macrophage resistance towards ferroptosis (16, 17). In this context, decreased Nrf2 activity might promote ferroptotic death of Lyve1+ resident macrophages at early stages of plaque development and in this way contribute to disease progression. This, however, would require further investigation.

In terms of bone marrow-derived macrophages, the role of Nrf2 is better recognized. Previous *in vivo* research demonstrated strong evidence that Nrf2 works as a negative upstream regulator of macrophage proinflammatory phenotype, while the majority of *in vitro* data indicates that Nrf2 deficiency promotes macrophage polarization towards proinflammatory M1 phenotype (25, 26, 31, 66). Macrophages are generally considered as non-dividing cells with limited capacity for self-renewal. However, our current data demonstrate that in Nrf2^Cdh5tKO^ proinflammatory macrophages the pathway associated with DNA replication was significantly enriched. The precise role of DNA replication in macrophage proliferation and activation (especially in atherosclerosis) is still not fully understood. There are indications that macrophages may undergo DNA replication and proliferation upon certain, usually considered as harmful or danger, stimuli (73, 74). The upregulation of *Atr* and *Apex1* in Nrf2^Cdh5tKO^ proinflammatory macrophages may suggest increased oxidative stress and DNA damage that often accompany Nrf2 deficiency (74). Some data indicate that high level of DNA synthesis aiming at DNA repair in macrophages is a response to oxidative DNA damage and is highly associated with apoptotic cell death (11). In chronic inflammation, DNA synthesis may raise generation of polyploid macrophages (73). In addition, we observed that Nrf2 deficiency in proinflammatory macrophages was associated with upregulation of genes from the CLR family, including Mincle and MCL, involved in lipids recognition (including cholesterol) and their endocytosis (75). Our transcriptomic analysis of proinflammatory macrophages from atherosclerotic Nrf2^Cdh5tKO^ mice also revealed decreased expression of genes connected with autophagy (*Mcm7*, *S100a4* and *Gpnmb*; **Table 5**) and genes described as ferroptosis-related (*Hells* and *Cp*; **Table 5**). Observations of the last years indicate that the process of autophagy and ferroptosis are intertwined (76). Autophagy promotes intracellular lipid hydrolysis and cholesterol efflux thereby inhibiting development of macrophage-derived foam cells. Inhibition of autophagy in macrophages leads to foam cells formation, macrophage death, pro-inflammatory factor release and contributes to atherogenesis (77). Autophagy plays a key role in inhibiting ferroptosis of macrophages *via* maintaining cellular iron homeostasis and cellular reactive oxygen species generation, and alleviates atherosclerosis (78, 79). Overall, our data indicate that Nrf2 deficiency in atherosclerotic proinflammatory macrophages affects the expression of genes involved in DNA replication and repair mechanisms, as well as in the recognition of lipids and cell death pathways, what may impact these cells activation, phagocytic ability, and survival.

In atherosclerotic conditions, Nrf2-deficiency caused transcriptomic changes also in the monocyte subset. Some proinflammatory genes were downregulated and anti-inflammatory were upregulated, whereas on the other hand, the expression of some chemoattractants was increased when compared to monocytes from the aortas of atherosclerotic Nrf2^flox/flox^ mice. The observed changes suggest modulation of the immune phenotype of monocytes, but not necessarily towards the proinflammatory one. In addition, in atherosclerotic Nrf2^Cdh5tKO^ *vs.* atherosclerotic Nrf2^flox/flox^ monocytes, increased level of *Mmp12* and protease inhibitors may suggest their involvement in ECM remodeling and monocyte infiltration.

In conclusion, this exploratory study identified several subtype-specific differences in monocytes and two selected, the most numerous in the aortic wall, types of macrophages from atherosclerotic Nrf2^Cdh5tKO^ mice. The introduced proatherogenic factors caused significant changes particularly in the expression of genes encoding inflammatory cytokines and chemokines. Comparison of atherosclerotic Nrf2^flox/flox^ and Nrf2^Cdh5tKO^ mice enabled identification of Nrf2-dependent macrophage subtype-specific transcriptomic changes associated with inflammation, iron homeostasis, DNA repair, cell injury and death pathways. Our data demonstrate a possible link between ferroptosis and inflammatory microenvironment appearing at a very early stage of atherogenesis. A limitation of this study can be our mouse model based on Cdh5-dependent Cre activity. First, because Nrf2-deficient endothelial cells may affect the microenvironment and their interaction with other cell types (e.g. monocytes) may differ. Second, this mouse model is characterized by a significant downregulation, but not a complete knockout of Nrf2 in mononuclear phagocytes. Although, we think that such model better reflects the observed in humans aging-associated decline of Nrf2 activity, in the future it may be worth to reproduce some of the findings in mice with the lysozyme 2 (LysM) Cre-driven recombination.

## Data availability statement

The data presented in the study are deposited in the Gene Expression Omnibus (GEO) repository, accession number GSE245820.

## Ethics statement

The animal study was approved by the 2nd Institutional Animal Care and Use Committee (IACUC) in Kraków, Poland. The study was conducted in accordance with the local legislation and institutional requirements.

## Author contributions

KSa and AJ-K designed the study. KSa, MS, IK, and KSz performed the experiments. MS and KSa performed the bioinformatic analyses and data visualization. KSa and AJ-K were involved in data interpretation and wrote the manuscript. JCS, PB, and JD provided the intellectual contribution and revised the manuscript. AJ-K secured funding and coordinated the project. All authors provided constructive feedback, helped shape the manuscript and approved the submitted version.
